# Idiopathic Membranous Nephropathy: Glomerular Pathological Pattern Caused by Extrarenal Immunity Activity

**DOI:** 10.3389/fimmu.2020.01846

**Published:** 2020-09-16

**Authors:** Wenbin Liu, Chang Gao, Zhiyuan Liu, Haoran Dai, Zhendong Feng, Zhaocheng Dong, Yang Zheng, Yu Gao, Xuefei Tian, Baoli Liu

**Affiliations:** ^1^Beijing Hospital of Traditional Chinese Medicine, Capital Medical University, Beijing, China; ^2^Basic Medical College, Taishan Medical University, Tai'an, China; ^3^Beijing Chinese Medicine Hospital PingGu Hospital, Beijing, China; ^4^Shunyi Branch, Beijing Hospital of Traditional Chinese Medicine, Beijing, China; ^5^Section of Nephrology, Department of Internal Medicine, Yale University School of Medicine, New Haven, CT, United States

**Keywords:** autoimmune response, inflammation, pathogenesis model, podocyte, spontaneous remission, environmental stimuli

## Abstract

Idiopathic membranous nephropathy (IMN) is a pathological pattern of glomerular damage caused by an autoimmune response. Immune complex deposition, thickness of glomerular basement membrane, and changes in the podocyte morphology are responsible for the development of proteinuria, which is caused by the targeted binding of auto-antibodies to podocytes. Several auto-antigens have recently been identified in IMN, including M-type receptor for secretory phospholipase A2 (PLA2R1), thrombospondin type-1 domain-containing 7A (THSD7A), and neural epidermal growth factor-like 1 protein (NELL-1). The measurement of peripheral circulating antibodies has become an important clinical reference index. However, some clinical features of IMN remain elusive and need to be further investigated, such as the autoimmunity initiation, IgG4 predominance, spontaneous remission, and the unique glomerular lesion. As these unresolved issues are closely related to clinical practice, we have proposed a hypothetical pathogenesis model of IMN. Induced by environmental stimuli or other causes, the PLA2R1 antigen and/or THSD7A antigen exposed to extrarenal tissues, such as lungs, then produce the auto-antibodies that target and cause damage to the podocytes in circulation. In this review, we highlighted the potential association between environmental stimuli, immune activity, and glomerular lesions, the underlying basis for spontaneous immune and proteinuria remission.

## Introduction

Membranous nephropathy (MN) is one of the major glomerular diseases in adults, accounting for 20–30% of glomerular disease cases (1, 2). MN is pathologically characterized by the accumulation of immune complex deposits outside the glomerular basement membrane (GBM), which is adjacent to podocytes. The thickening GBM appears to have a “spike” to enfold the deposits (3). Immune complex deposits contain the antigens *in situ*, immunoglobulins (Ig) G binding to antigens, and membrane attack complexes (MAC) formed by complement activation, which are the traces left by the antibody-dependent immune response and the major basis for the current understanding of the pathogenesis of MN (4). This unique glomerular lesion is thought to be associated with many causes. According to patient histories and clinical manifestations, about 20% of MN can be attributed to clinical diseases, such as hepatitis B infection, systemic lupus erythematosus, cancer, or drug side-effects, which are known as secondary MN. In addition, about 80% of MN are clinically unable to be identified by the secondary factors, which are known as idiopathic or primary MN (IMN or PMN) (4). IMN is thought to be caused by IgG that target podocytes; the predominant form of IgG is considered to be IgG4 (5, 6). The podocyte auto-antigens identified in adult IMN include M-type receptor for secretory phospholipase A2 (PLA2R1) (7), thrombospondin type-1 domain-containing 7A (THSD7A) (8), and neural epidermal growth factor-like 1 protein (NELL-1) (9), which accounts for 70–80, 3–5, and 5–10% of IMN, respectively. Moreover, the measurement of antibodies against PLA2R1 (aPLA2R1ab) in peripheral blood has been widely used in clinical diagnosis (10), prediction, and treatment guidance (11). The natural history of the untreated MN is variable; spontaneous remission occurs in 40–50% of patients and the remaining patients progress slowly to end-stage renal disease (ESRD) or decease due to complications or any other unrelated underlying diseases after 5–15 years (12). IMN recurs in about half of kidney allograft recipients, which leads to graft dysfunction and failure (13).

The understanding of the pathogenesis has been improving in the last two decades (14). However, many factors in IMN still need to be further clarified. For instance, how does the immune response initiate the produce of the auto-antibodies against podocytes? Why are the auto-antibodies predominately IgG4? And how does the spontaneous remission occur in some patients? This review attempts to propose a hypothetical pathogenesis model to answer these issues while summarizing the main findings from the relevant available literature.

## Extrarenal Autoimmune Response Can Cause IMN

IMN is mainly considered to be an organ-restricted autoimmune glomerular disease due to the discovery of podocyte auto-antigens such as PLA2R1 (3). However, there is currently a lack of evidence that IMN is an autoimmune disease induced by *in situ* podocyte auto-antigens. In addition, Xu et al., van de Logt et al., and our team have recently proposed the hypothesis of IMN pathogenesis induced by an extrarenal autoimmune response (mainly in the lungs) (14–16). Despite the current lack of direct evidence, we can speculate on the feasibility of this hypothesis from the following topics.

### Circulating Antibodies Against Podocyte Exteriors of the Kidney Cause IMN Development: Hints From Previous Studies

Heymann nephritis, the first experimental MN induced in rats, was described in 1959, and was then known as active Heymann nephritis (AHN), which was immunized by an insoluble subcomponent of the brush border of the proximal tubule, called fraction 1A (Fx1A). Subsequently, it was found that injection of anti-Fx1A IgG in rats could induce the analogous lesion more rapidly, which was named as passive Heymann nephritis (PHN) (4). In 1978, two different teams from Couser and Hoedemaeker infused anti-Fx1A IgG into isolated rat's kidneys *ex vivo* to study pathological changes of IMN; the idea that extrarenal circulating antibodies bind podocyte auto-antigens to form *in situ* immune complexes was proposed first (17, 18). Later, the major antigenic proteins in Fx1A were identified as megalin, which is a podocyte membrane receptor of low-density lipoproteins (19, 20). IgG passes through the glomerular endothelial cells and GBM freely, thus binding to megalin on the surface of podocytes, which in turn forms PHN (21). Other experimental MN induced by injection of exogenous anti-podocyte antibodies were carried out, such as a mice model of anti-podocyte glomerulonephritis, THSD7A homologous rodent MN, and transgenic mice MN with PLA2R1, which are similar to that of PHN (22–24). These findings suggest that extrarenal circulating antibodies against podocytes can induce MN-like pathological changes in normal kidneys. And, this phenomenon is not only limited to experimental MN.

In 2002, the neutral endopeptidase (NEP) was identified by Ronco's team in neonatal MN (25); it supports the idea that the circulating anti-podocyte antibodies can be produced without the involvement of the kidney. Mothers with NEP deficiency were exposed to NEP during a previous spontaneous abortion or pregnancy, and their immune systems produced anti-NEP antibodies afterwards, which crossed the placenta barrier and bound to the fetal glomerular podocytes during pregnancy (26). Similar to PHN and other above-mentioned experimental MN models, nephritogenic antibodies of neonatal MN are produced not by the person themselves but by the immune system of nonego, which can be classified as “passive MN.” These previous studies suggest that IMN can be induced by an autoimmune response outside the kidney.

### Potential Relationship Between Extrarenal Disease, Environment, and Pathogenesis of IMN

After the identification of auto-antigens such as PLA2R1, the pathogenesis of IMN was considered as primary MN (3). However, when idiopathic and secondary MN are distinguished using circulatory aPLA2R1ab, its diagnostic specificity is relatively inadequate. It was reported that 9 out of 32 patients with secondary MN presented with positive anti-PLA2R1, including 7 cancer patients, 1 Crohn's disease patient, and 1 patient with scleroderma (27). Furthermore, the result from another cohort showed that, among the 24 patients with psoria-associated MN, 7(29.2%) patients had positive anti-PLA2R1 (28). And, in 39 patients of hepatitis B virus-associated MN, 25(64.1%) patients with aPLA2R1ab positive were found (29). Also, among the 37 patients with membranous lupus nephritis, 7(18.9%) patients were found to be anti-PLA2R1. More interestingly, 3 patients with non-renal lupus disease were found to be anti-PLA2R1 positive (30). There is a possibility that PLA2R1-associated MN coexists with these extrarenal diseases, as the causal relationship between them cannot be completely excluded (31).

The causal relationship in THSD7A-associated MN is more obvious. Although the association between THSD7A and cancer is disputed (32, 33), THSD7A can be detected in the tumor, metastatic lymph node cells, and dendritic cells of lymph nodes (34, 35). In a cohort of breast and colorectal tumor patients, THSD7A is also expressed in breast tumor tissues (20/20, 100% patients positive) and colorectal tumor tissues (79/81, 97.5% patients positive). More importantly, in 3 of the 4 tumor patients with proteinuria who were followed up, proteinuria was in complete remission within 6 months after tumor resection without any other interventions (36). However, the kidney disease of these 4 patients were undiagnosed, even though THSD7A is currently considered only associated with IMN. A patient who had repeated renal biopsy showed negative expression of THSD7A in their kidney tissue in the first biopsy, however the second renal biopsy showed the MN lesion with positive THSD7A and manifested malignant tumor after 17 months. After 4 months, the serum THSD7A auto-antibody showed negative after surgical resection and chemotherapy with paclitaxel liposome carboplatin (37). More interestingly, inspired by two cases of THSD7A-associated MN accompanied by angiolymphoid hyperplasia with eosinophilia (ALHE), the researchers assumed that vascular endothelial growth factor A (VEGF-A) up-regulated the expression of THSD7A in endothelial cells *in vitro* (38). The tumor growth is closely related to angiogenesis; VEGF is secreted by cancer cells and stromal cells stimulate the proliferation and survival of endothelial cells, leading to the formation of new blood vessels (39). This may be one of the ways for THSD7A to establish the relationship between tumor and IMN. Extrarenal diseases may induce IMN by affecting these auto-antigens expressed outside the kidney. The autoimmune response of IMN (or primary MN) may not be limited to the kidneys, when precisely observed.

The pathogenesis of IMN has been associated with environmental stimuli (40). The morbidity of MN in China has been gradually increasing, which could be possibly related to the long-term exposure to air pollution. Each 10 mg/m^3^ increase in PM2.5 concentration over 70 mg/m^3^ is related with 14% higher odds for MN (2). It is speculated that air pollution can be involved in the pathogenesis of IMN by inducing inflammation and oxidative stress in the lungs (14–16), it can also activate antigen presenting (APC) cells and autoreactive T cells in the inflammatory microenvironment. In addition, helicobacter pylori (HP) is closely related to chronic mucosal inflammation in the stomach and duodenum (41), and MN patients are also closely related to gastric HP infection (42–44). Consistent with these speculations, two risk alleles for primary MN have been recently identified in *NFKB1* and *IRF4*, and both of them were involved in defense against common pathogens. It is worth mentioning that all four genome-wide significant risk loci (*PLA2R1, IRF4, NFKB1, and HLA*) with highly pleiotropic effects identified in this extensive genetic study have a concordant effect on the risk of inflammatory bowel disease (IBD); this fact suggests that there is a shared pathogenic mechanism between IBD and IMN (45). Thus, inflammation can be one of the pathways for environmental stimuli and extrarenal diseases, which links to the pathogenesis of IMN.

### Non-inflammatory Glomerular Lesion of IMN Might Not Be Consistent With *in situ* Induced Organ-Specific Autoimmune Diseases

Unlikely most organ-specific autoimmune diseases induced by *in situ* auto-antigen exposure, such as diabetes mellitus type 1, inflammatory bowel disease (IBD), multiple sclerosis, and psoriasis, in which affected organs are infiltrated with immune cells (46–49), the kidney pathological manifestations of IMN shows almost no inflammatory cells' infiltration except for complement activation (4). As mentioned above, the pathogenesis of IMN is probably associated with inflammation, and it is difficult to imagine an autoimmune response induced by auto-antigen exposure in glomerulus without any involvement of immune cells. There is a hypothesis that a soluble form of podocyte auto-antigen, such as soluble PLA2R1, sheds and induces an autoimmune response (4), which could partly explain the non-inflammatory lesion to the glomerulus. However, direct evidence of an autoimmune response induced by soluble PLA2R1 is still lacking, and this hypothesis is poor. Firstly, it is unlikely that podocytes will release large amounts of soluble PLA2R1 without being affected. If so, the presence of proteinuria should predate the autoimmune response. Secondly, soluble PLA2R1 should be distributed in the blood vessels according to the concentration gradient; the closer one goes to the podocytes, the higher the concentration, which also possibly induces the immune cells to approach the glomerulus and deposit circulating immune complexes at multiple sites in the glomerulus. IMN caused by an extrarenal autoimmune response can be a better explanation for these kidney pathological manifestations. In addition, the deposition of extrarenal IgG4 also further inhibits the inflammation in the glomerulus (detailed below).

### Auto-Antigens Associated With IMN Pathogenesis Expresses in Many Tissues Including the Kidneys

Besides the kidney podocyte, PLA2R1 also express in the lungs, placenta, liver, and skeletal muscles (50). PM2.5 induces inflammatory responses in the lungs, causing infiltration of inflammatory cells (51, 52), such as neutrophils and alveolar macrophages, which also express PLA2R1 (53, 54). The extrarenal expression of PLA2R1 provides the basis for extrarenal aPLA2R1ab production. Interestingly, the production of aPLA2R1ab does predict significant kidney damage. Recently, Burbelo et al. reported that aPLA2R1ab were detectable at a median of 274 days before renal biopsy diagnosis (interquartile range, 71–821 days) and aPLA2R1ab seropositivity occurred closely or before the pre-diagnostic non-nephrotic range proteinuria in the majority of cases (55). The aPLA2R1ab is considered directly pathogenic and the kidneys can just be a target. The direct evidence of humoral immunity induced by exposure to the lung's PLA2R1 is required for further studies.

THSD7A was initially characterized as an endothelial protein that is expressed in the placental vasculature, which can be involved in promoting endothelial cell migration during angiogenesis (56–58). Angiogenesis mostly occurs in tissue repair or tumor metastasis, and in this phenomenon, both inflammation and active immune response are down-regulated, which is also consistent with the immunosuppressive effect of VEGF (39). The immune induction phenomena of THSD7A seems to be different from that of PLA2R1.

NELL-1 is a newly discovered third auto-antigen of IMN podocytes (9), which is highly expressed in osteoblasts and promotes bone regeneration. The C-terminal region of NELL-1 mediates osteoblastic cell adhesion through integrin α3β2 (59, 60). NELL-1 is overexpressed in patients with craniosynostosis, one of the most common congenital craniofacial deformities, where it is specifically up-regulated within prematurely fusing sutures (61, 62). It indicates that different auto-antigens in IMN have different expression phenomena, and these different phenomena that affect the exposure of auto-antigens are potentially mutually exclusive. IMN may be the same pathological pattern of different diseases. Thus, it is necessary to figure out that different phenomena of different auto-antigens initiating extrarenal autoimmune response leads to IMN.

## Auto-Antigens and Extrarenal Autoimmune-Inducing Phenomena

The autoimmune response is the result of a combination of factors, and the fundamental question regards how self-tolerance fails and how self-reactive lymphocytes are activated. Despite the lack of research on these topics in IMN, we can still try to figure out some important questions on the basis of our current understanding.

### Characteristics of Auto-Antigens

One of the key preconditions for an extrarenal autoimmune response that involves the kidney is that these antigenic proteins must be expressed in the kidney and, more precisely, in the podocytes. Virtually all structures in the glomerulus and all domains and micro-domains of the endothelium and podocyte are accessible to the circulating antibodies. More importantly, the fate of immune complexes formed by binding to glomerular components varies with the location of the glomerulus antigens, the basal domain, and/or the slit diaphragm, which persists longer than the others (63). This finding may suggest that the location of auto-antigenic proteins may determine their ability to cause damage to podocytes. Recently, using a variety of microscopes, it was found that THSD7A localizes at the basal aspect of foot processes, closely following the meanders of the slit diaphragm in human and mice, and the anti-THSD7A antibodies bind THSD7A expressed on the slit diaphragm (64). In human podocytes, THSD7A expression is accentuated at filopodia and thin arborized protrusions, an expression pattern associated with the decreased membrane activity of cytoskeletal regulators. Phenotypically, THSD7A expression in human podocytes is associated with an increase in cell size and enhanced adhesion to collagen type IV-coated plates (64). Again, due to the lack of evidence on the precise localization of PLA2R1, it is currently reasonable to believe in renal pathology that PLA2R1 could also express at the basal domain or slit diaphragm of podocytes and can disrupt the function of podocytes, such as their adhesion ability to collagen type IV (GBM) (65). The uniformity of NELL-1 staining along the GBM and correlation with the subepithelial deposit suggests that this protein is shed from the podocytes (9). It is unlikely that NELL-1 is shed from mesangial cells or endothelial cells, since there was no mesangial or subendothelial staining in the NELL-1 positive MN. Nevertheless, NELL-1 is also likely present as an extracellular component and can deposit in the GBM (9). These antigens are restricted to express in the space between podocytes and GBM, which could also be the reason for the limitation of pathological manifestations.

Another key precondition is that the autoimmune-inducing phenomena outside the kidney must specifically induce exposure of these antigens, such as conformational changes, molecular simulations, or up-regulated expression. From the mutual exclusion of these antigenic proteins in IMN, it seems that the immune-inducing environment of different antigens is also mutually exclusive. Interestingly, the subclass of IgG deposition of NELL-1-associated MN is mainly IgG1, which is inconsistent with PLA2R1-and THSD7A-associated MN with the IgG4 as the principal antibody (9). It suggests that there is heterogeneity in the autoimmune process of IMN, and needs to be further subdivided according to different immune processes in future studies. In other words, the variety of auto-antigens can be selected by different immune-induced phenomena.

### PLA2R1-Autoimmune Response, Inflammation, and Genetic Susceptibility

**Figure 1:**
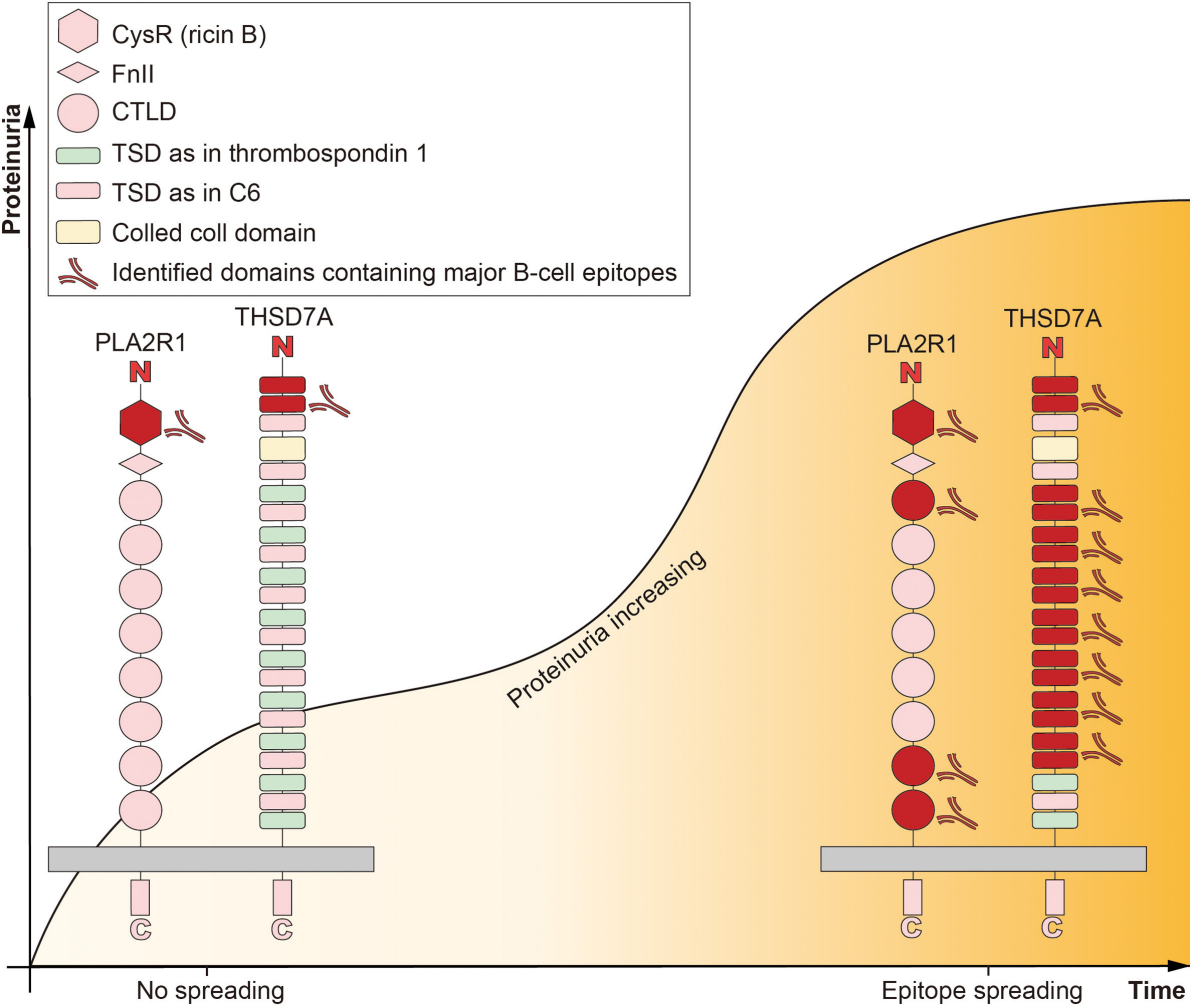
Epitope spreading accompanied with diseases progression in idiopathic membranous nephropathy. The extracellular part of M-type receptor for secretory phospholipase A2 (PLA2R1) is composed of N-terminal cysteine-rich (CysR, or ricin B) domain, a single fibronectin type II (FnII) domain, and 8 C-type lectin-like domains (CTLDs). The four identified domains containing major B-cell epitopes are CysR, CTLD1, CTLD7, and CTLD8. CysR is the predominant target of autoimmunity. The extracellular part of thrombospondin type-1 domain-containing 7A (THSD7A) is composed of a colled coll domain and 21 thrombospondin type 1 domains (TSDs). These TSDs show high structural homology either to the TSDs of thrombospondin 1 or complement component 6 (C6). The predominant target of autoimmunity is exists in the N-terminal region. Epitope spreading is defined as the diversification of epitope specificity from the initial focused, dominant epitope-specific immune response, to subdominant and/or cryptic epitopes, which accompanied by maturation of the immune response and progression of the disease (proteinuria increasing).

PLA2R1 belongs to the mannose receptor family and is a type I transmembrane glycoprotein with 185kDa, whose extracellular part is composed of N-terminal cysteine-rich (CysR, or ricin B) domain, a single fibronectin type II (FnII) domain, and 8 C-type lectin-like domains (CTLDs) (66). The four identified domains containing major B-cell epitopes are CysR, CTLD1, CTLD7, and CTLD8, and epitope antigenicity in these domains is all determined by spatial conformation (67, 68). The N-terminal region of PLA2R1 is the predominant target of autoimmunity, as is THSD7A, and epitope recognition evolves over time and as the disease worsens (69–71). Patients with epitope spreading usually have more severe disease and worse prognosis (Figure 1). In general, PLA2R1 acts as the receptor mediating sPLA2 endocytosis, continuously trafficking between the cell membrane and the endosome to transport sPLA2 (50, 66, 72). Beyond mediating sPLA2, the current understanding of the biological function of human PLA2R1 is quite limited. It is speculated that human PLA2R1 is closely related to inflammation.

Either through ovalbumin-induced lung inflammation or murine α-myosin heavy chain-induced autoimmune myocarditis, the affected tissues in the PLA2R1-deficiency mice show more inflammatory cell infiltration and more severe inflammatory response (73, 74). Furthermore, rat PLA2R1 expression was up-regulated in an inflammatory environment (for example, PLA2R1 expressed on lymphocytes and granulocytes can be up-regulated by interleukin (IL)-1b *in vitro*) (75). However, these studies have limitations since the rodent kidney does not express PLA2R1. Human PLA2R1 can also be involved in inflammatory processes, such as the potential function of PLA2R1 expressed on neutrophils and alveolar macrophages (53, 54). Human PLA2R1 is also closely related to asthma. PLA2R1 is differentially overexpressed in bronchial epithelial brushings of children with atopic asthma (76); it is also believed that there could be a potential involvement of risk alleles on *PLA2R1* in adults with asthma who have occupational exposure, which plays an important role in NF-κB pathway, leading to inflammation (77). We hypothesized that human PLA2R1 can be up-regulated in an inflammatory environment, which can be related to the autoimmune-induced environment of PLA2R1 in IMN.

Genome-wide association studies (GWASs) clarified the understanding of IMN genetics. The two risk alleles in *HLA-DQA1* and *PLA2R1* are closely related to IMN development, and the same results have been obtained in multiple interracial genetic studies (45). Recently, *NFKB1* and *IRF4*, the novel genome-wide significant risk loci for MN have been identified, with large effects encoding two transcriptional master regulators of inflammation. Due to the bi-ethnic composition of the cohort, the ethnicity-specific effects at the HLA locus can be further defined. DRB1^*^1501 is a major risk allele in East Asians, DQA1^*^0501 in Europeans, and DRB1^*^0301 in both ethnicities, which suggests that different epitopes are likely presented to T cells to initiate the anti-PLA2R1 response in East Asians and Europeans. In line with previous studies, a single haplotype at the PLA2R1 locus exhibits genetic interactions with HLA-DRB1 risk alleles in both ethnicities, the three amino acid residues encoded by DRB1^*^1501 and DRB1^*^0301 in the DRβ1 chain of major histocompatibility complex (MHC) class II promoting epitope presentation to T cells, which could be the cause of IMN susceptibility (45, 78).

Under the activation of antigenic peptides and MHC class II molecules, CD4^+^ T cells differentiate into several subtypes. This process is regulated by many cytokines produced by innate immune cells. Previous studies have observed that IMN is dominated by Th2 type immune response with increased IL-4 production of peripheral Th cells, and increased IL-13 mRNA expression of peripheral blood monocytes in patients (79, 80). Rosenzwajg et al. and Roccatello et al. described a low level of regulatory T (Treg) cells in two cohorts of IMN (*n* = 25 and *n* = 17, respectively) at diagnosis and an increasing level of these cells after remission induced by rituximab, associated with low levels of a Treg cytokine IL-35 that also increases with remission (81, 82). Although the effector cells that induce T cell differentiation in IMN and their immune-induced microenvironment are not yet clear, the above data also suggest an inflammation tendency in IMN.

## IgG4 Dominate and the Role of IgG4

IMN is considered an IgG4-dominant disease (7, 8). In three IMN cohorts of detected peripheral circulating IgG subclasses, all the positive rates of IgG4 were the highest, with the proportions of 89% (83), 94% (84), and 100% (85), respectively, and the titer of IgG4 was also the highest. Among other IgG subclasses, IgG1 also accounts for a higher percentage in IMN patients, but the titer is relatively insufficient (84). Interestingly, the deposited IgG subclass in the glomerulus indicates the Ig subclass switch as the disease progresses. In the early stage (electron microscopy stage I), IgG1 is the major IgG subclass, and IgG4 dominates in all later stages (electron microscopy stage II-IV) (by pathological criteria according to Ehrenreich and Churg, IMN is divided into four stages) (86). Correspondingly, IgG1, IgG2, IgG3, and IgG4 coexist in most patients at the first stage of the disease, while at relapse, IgG4 was the predominant subclass (87). When comparing with other IgG subclasses, IgG4 is usually unable to activate complement and has a higher affinity for antigens (88, 89). Exostosin 1 (EXT1)/exostosin 2 (EXT2)-associated MN represent a secondary form of MN and the antibody in EXT1/EXT2-associated MN it is IgG1 (90). Ravindran et al. described a high spectral count of complement proteins C3, C4, C5, C6, C7, C8, and C9 in glomerular that both exist in PLA2R-associated and EXT1/EXT2-associated MN by using mass spectrometry. However, complement protein C1 was present in low spectral counts in EXT1/EXT2-associated MN, which appeared higher than in PLA2R-associated MN (91). Each of the three complement pathways seem to be involved in the glomerular complement activation, while classical pathways may not be evident when IgG4 is dominant (87, 91), which suggest that the respective antibody titers and ratios between the different IgG subclasses will determine the involvement of different pathways of complement activation. Thus, it is necessary to discuss the potential causes of Ig class switching of antibodies in IMN.

### Reasons for IgG4 Domination: Anti-inflammatory and Increased Affinity

The diversity of the human antibody repertoire is generated by V(D)J gene rearrangement that gives antibodies their complex array of effector functions, and accumulation of VDJ somatic point mutations are generally considered to boost higher affinity antibodies through selection within the germinal centers (88). IgM > IgG3 > IgG1 > IgG2 > IgG4 is considered to be the temporal model sequence of Ig class switching in the germinal center reaction. Differences in epitopes' affinity, complement fixation ability, and Fc receptor (FcR) binding ability between these subclasses could help to coordinate the inflammation and humoral defenses over the time course of a response (88). At the early stage of germinal center reaction, IgM^+^ B cells switch to IgG3, which recruits FcγR-mediated functions in the early response. IgG1 then emerges as the major effector of antigen clearance, and subsequently IgG2 competes with IgG1 to produce immune complexes which slows the inflammatory drive. Persisting antigens can finally stimulate high affinity IgG4 that outcompetes other isotypes (88, 92). IgG4 may inhibit the binding of other IgG subclasses to antigens by its high affinity, reaching a certain proportion in the formed immune complex and blocking FcyR-mediated processes, such as phagocytosis and release of pro-inflammatory cytokines (88). IgG4 response is often formed by following repeated or long-term exposure to antigen (89, 93). For instance, IgG4 plays a protective role in allergies, with prolonged or repeated allergen exposure and increased production of IL-10, and IgG4 gradually increases and blocks IgE to inhibit excessive allergic reactions (94). And, in asymptomatic filarial infection, elevations in IgG4 are also often associated with high worm loads and with high plasma levels of IL-10 (95). As a key cytokine for immune regulation, IL-10 plays an important role in inhibiting the overreaction to antigens, including inflammation and adaptive immune responses (96). IL-10 is also considered to specifically modulate the differentiation of B cells into IgG4-responsive types (97, 98). Up-regulation of IL-10 mRNA expression was observed in peripheral blood monocytes in IMN patients (80). During the early stage of IMN, patients have higher counts of M2-like monocytes, higher levels of serum IL-10, and the IL-10^+^ M2 counts were positively correlated with disease activity (99), which could suggest that antibody class switching is driven by innate immunity in the early stage of IMN. In general, the anti-inflammatory properties of IgG4 and its high affinity to antigens are responsible for its delay appearance and dominant role in the active immune process (88, 89, 93).

### Hypothetical Model: Ig Class Switching of Antibody and IMN Disease Progression

As mentioned above, IMN is closely related to inflammation, environmental stimuli, and genetic susceptibility. Pathogens or PM2.5, as the continuous stimulus, often induce constant local chronic inflammation. It also leads to the accumulation and continuous activation of macrophages at the inflammatory site, so as to cause bystander damage (100–102). In inflammatory conditions, auto-antigenic proteins can induce autoimmune diseases by altering conformation and epitopes, dissociating from tissues, up-regulating expression, or further modifying auto-antigens, while exposed to the immune system (103–105). Even the inflammatory cells themselves can be the sources of auto-antigens (both neutrophils and alveolar macrophages expressing PLA2R1) in IMN (14). The exposure of autoantigens then aggravates the local inflammation, which leads to the formation of a vicious circle (105). Under the stimulation of environmental factors, the function of immune cells will also be affected, such as the auto-reactive T cells and the antigen presenting cells, thus increasing the risk of loss of self-tolerance abilities (106, 107). This will subsequently induce the humoral immunity targeted to the extrarenal auto-antigen (PLA2R1 or THSD7A), which is programed to control the local inflammation and tissue damage.

**Figure 2:**
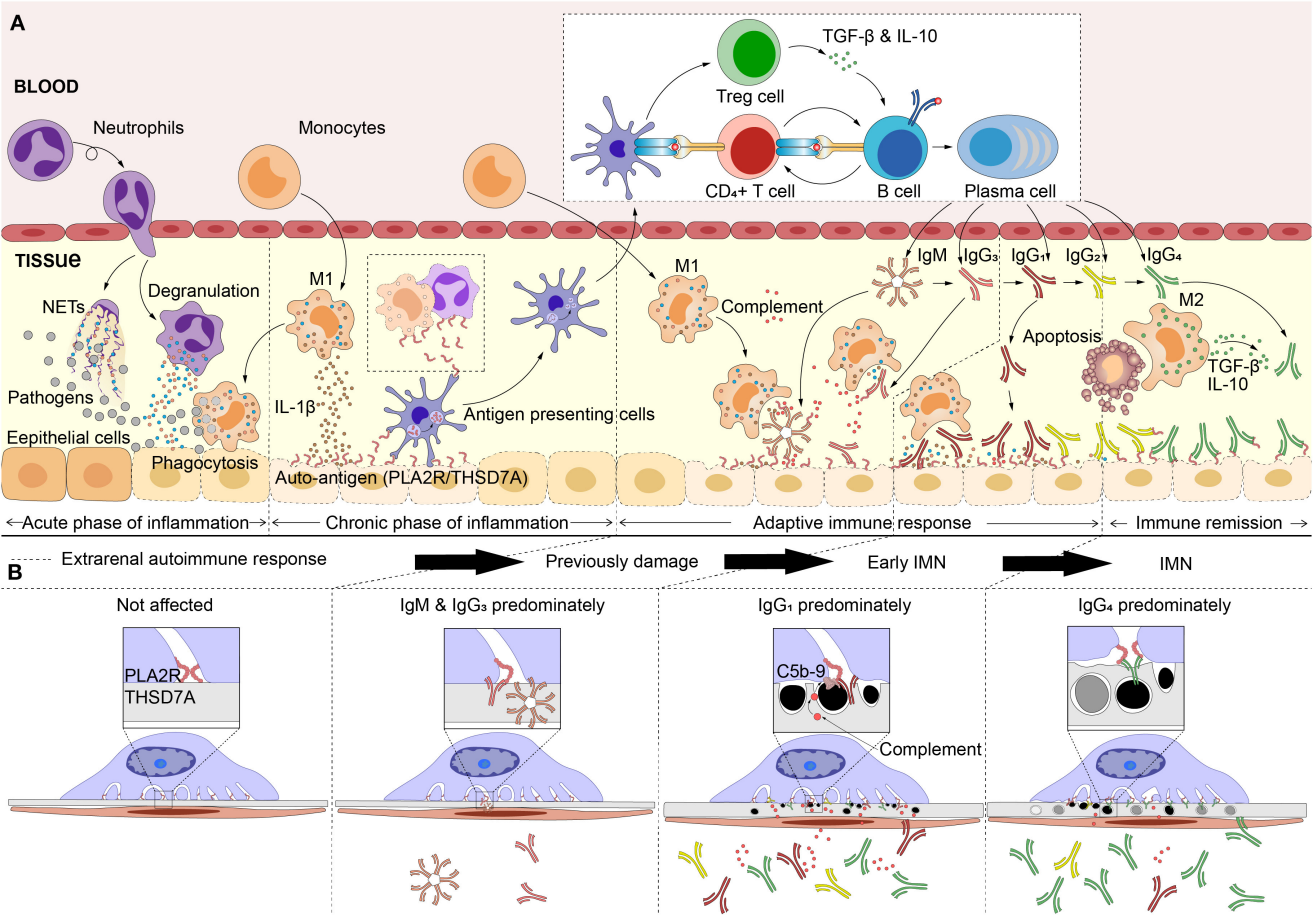
Hypothetical pathogenesis model of idiopathic membranous nephropathy. **(A)** The pathogens (inhalable particles, bacteria and virus, etc) or other causes that affects the extrarenal tissues inducing inflammatory response. As the stimulus continues, the inflammation gradually goes in the phase that is dominated by M1 macrophages (encourage inflammation), and causes tissue damage (bystander damage). Hence, the auto-antigens such as M-type receptor for secretory phospholipase A2 (PLA2R1) or thrombospondin type-1 domain-containing 7A (THSD7A), are exposed in extrarenal inflammatory microenvironment. Pathogens or inflammation enhance the immunogenicity of autoantigen and affect the antigen processing capacity of antigen presenting cells, which leads to the auto-immune response. With the maturation of antigen affinity of B cells and the induction of cytokines, the immunoglobulins (Ig) undergo the class switching follow this sequence: IgM> IgG3> IgG1> IgG2> IgG4. IgM, IgG3, or IgG1, which can activate the complement system and Fc receptors, leading to tissue inflammation, and damage. Subsequently, IgG2 competes with IgG1 to slow the inflammatory drive. Normally, after neutrophils or macrophages eat debris/pathogens they perform apoptosis and are removed by M2 macrophages (decrease inflammation and encourage tissue repair), which produces high levels of IL-10 and transforming growth factor (TGF)-β that promote inflammation remission. IL-10 and TGF-β that produced by M2 macrophages or Treg cells induced the IgG4-dominated immune response. **(B)** The antibodies which are produced, bind to the podocyte through circulation, resulting in idiopathic membranous nephropathy (IMN). Anti-PLA2R1/THSD7A IgM or IgG3 could have been deposited in glomeruli with very small quantities, induced the previously glomerular damage. However, the low affinity makes it difficult for them to bind to the antigens naturally expressed in podocytes for a long time. Subsequently, large deposits of IgG1 induced early IMN (stage I). IgG4 can competitively inhibits IgG1 binding to antigens with its higher affinity and achieving a major proportion in the immune complex, finally result in IMN.

Based on this understanding, we have further speculated about the development of IMN in this section, although it remains just a hypothesis. In the very early stages of IMN, anti-PLA2R1/THSD7A IgM or IgG3 could have been deposited in glomeruli in very small quantities. Since the source of the immune response is not present in the kidney, only very small quantities of IgM or IgG3 and the subsequent potential complement activation could cause previous damage to the podocytes. However, their low affinity makes it difficult for them to bind to the antigens which are naturally expressed in podocytes for a long time. Separate from IgM and IgG3; IgG1 has a high affinity for antigens, when the antibody subclass switch to IgG1 predominantly, the IgG1 in plasma can deposit in the glomerulus in large quantities, inducing early IMN (stage I). The original purpose of IgG1 production is to drive the inflammation and antigen clearance in the extrarenal site, while the immune system also aims to block the excessive inflammatory response simultaneously, resulting in the production of IgG2 and IgG4 (88, 89). IgG4 achieves bi-specificity through its Fab exchange, which can make a high-efficiency, high-affinity blocking antibody, inhibiting the excessive inflammation (108). Moreover, IgG4 can competitively inhibit IgG1 binding to antigens with its higher affinity, thus achieving a major proportion in the immune complex of IMN. With the deposition of IgG4, the glomerulus lesion aggravates. Although the complement activation is significantly reduced, the high affinity of IgG4 also can affect the normal function of podocytes, causing significant destruction (Figure 2).

### Potential Role of IgG4: Non-inflammatory and Organ-Limited Renal Manifestations

The anti-inflammatory properties of the autoimmune response that targets extrarenal sites could be the potential cause for the non-inflammatory lesions of IMN. Driven by extrarenal autoimmunity, IgG4 deposition is predominately found in the glomerulus of most IMN patients with its high production and affinity, which suppresses the inflammatory response. In addition, the immune complex of IMN is mainly subepithelial deposited, without direct contact with the blood. Even with the activation of complement to produce anaphylatoxins, such as C3a and C5a, it is difficult to cross the GBM and endothelial cells in reverse to induce a local inflammatory response. The elevated plasma levels of C3a and C5a in IMN patients (109) are more likely to be induced by extrarenal autoimmunity. Hence, neither plasma C5a and C3a are associated with IMN disease activity, however urinary C5a has a positive correlation with between plasma aPLA2R1ab levels and proteinuria (109). Thus, the products of subepithelial complement activation of glomeruli are more released into urine and do not play a role in inducing inflammatory cell infiltration.

**Figure 3:**
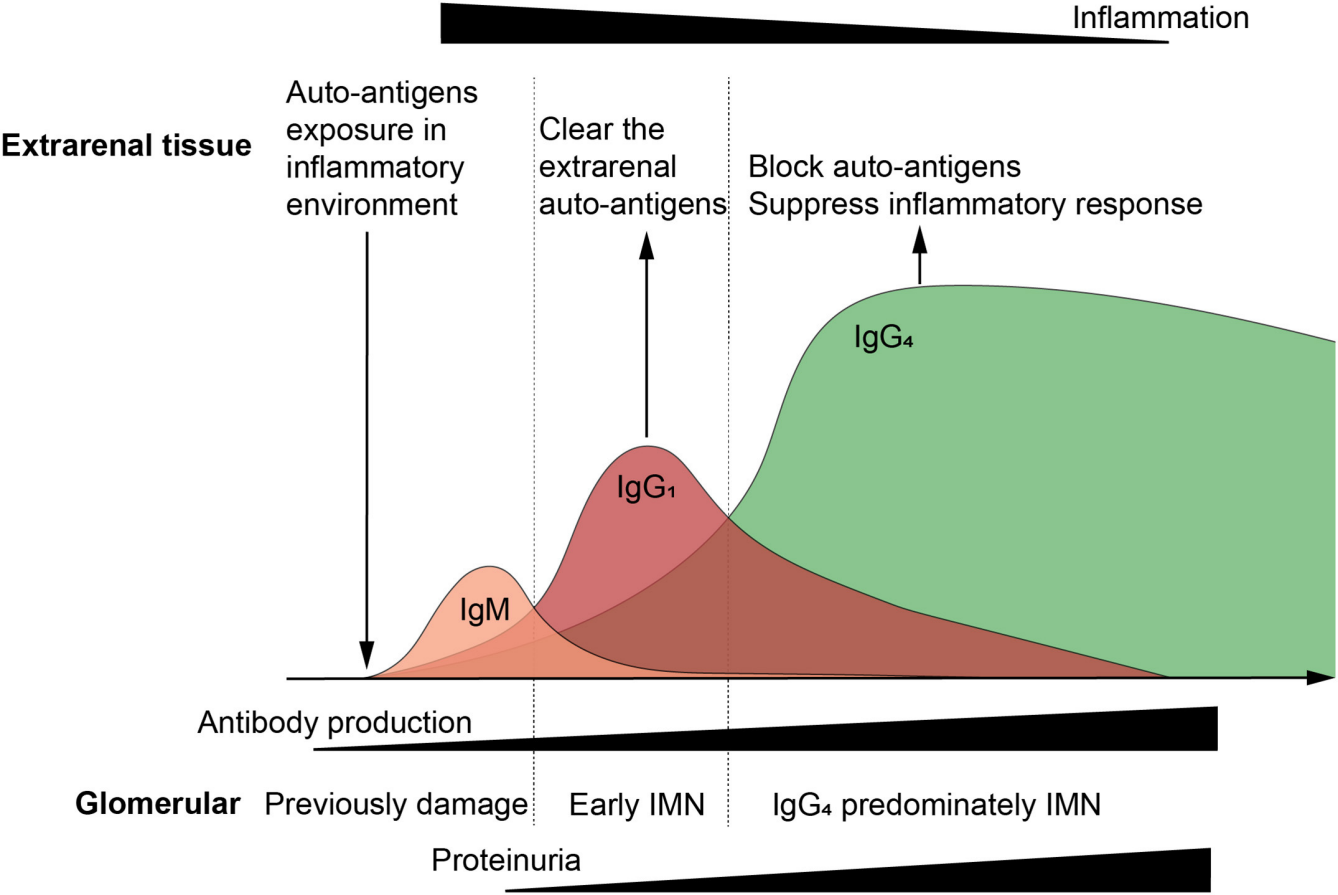
Relationship between extrarenal tissue, immune response, and glomerular damage. Auto-antigens exposure in inflammatory environment induce the auto-immune response, and subsequently immunoglobulins (Ig) undergo class switching. IgG1 mediates the most auto-antigen clearance as the primary effector antibody, and IgG4 blocks auto-antigens as the high affinity anti-inflammatory antibody. When pathogens and auto-antigens are effectively eliminated, the extrarenal inflammatory, and immune responses tend to be alleviated. However, large amounts of antibodies are deposited in the glomeruli, especially IgG4, leading to filtration barrier damage and proteinuria. Extrarenal remission and glomerular damage aggravation were observed, which can be the reason that why idiopathic membranous nephropathy is having trouble finding the primary foci.

A specific time period is required for extrarenal inflammation to induce autoimmunity and antibody affinity maturation. Included, repeated, or long-term exposure to antigens during this period can affect time duration. Without a certain affinity of antibodies, it is difficult for large amounts of IgG to be deposited due to the glomerular charge barrier. It will take time for the kidneys to present significant symptoms. Thus, the development of extrarenal tissue damage is prior to IMN progression and when large amounts of IgG4 are produced, the excessive extrarenal inflammation should also be suppressed, which can result in a time lag of symptoms in the extrarenal tissue, as well as in the kidney (Figure 3). More importantly, auto-antigens may function differently in different tissues (their function in extrarenal tissue is not as prominent as it is in the podocytes) and the symptoms of loss of function are not as specific as in the kidney, and it is difficult to establish a clear link clinically. The reported relationship between sarcoidosis and MN seems to support this argument; a high prevalence of PLA2R1-associated MN among patients with MN associated with active sarcoidosis has been described (31).

## The Spontaneous Remission of IMN

In the natural course, 40–50% of MN patients achieved spontaneous remission (12). Although spontaneous remission was more common in IMN patients with less baseline proteinuria, spontaneous remission also occurred in 22% of patients among those with baseline proteinuria >12 g/24 h (110). Immune remission predicts the remission of proteinuria, such as the decrease of aPLA2R1ab titer is earlier than that of proteinuria (111).

### Self-Termination of the Extrarenal Autoimmune Response: Immune Remission

The high affinity and anti-inflammatory properties of IgG4 can contribute to shaping the pathology of IMN glomeruli and inhibit the activity of the extrarenal autoimmune response. IgG4 binds to antigens in large quantities by its high affinity, blocks the immunogenicity of antigens, and competitively inhibits the proinflammatory activity of other IgG subclasses (88). In the case of antigen blocking and inflammation suppression, the immune response tends to be alleviated. The extent to which extrarenal auto-antigens are exposed to the immune system gradually decreases with IgG4 shows a dominant type of response. The immune remission of extrarenal sites is affected by multiple factors that are related to the degree of environmental stimulation and immune regulation, and the achieved degree of remission of individuals. With the gradual remission of the autoimmune response, the production of anti-podocyte antibodies decreases, and the deposited immune complexes are gradually degraded with the glomerular self-repair.

### Reversibility of Podocytes: Proteinuria Remission

**Figure 4:**
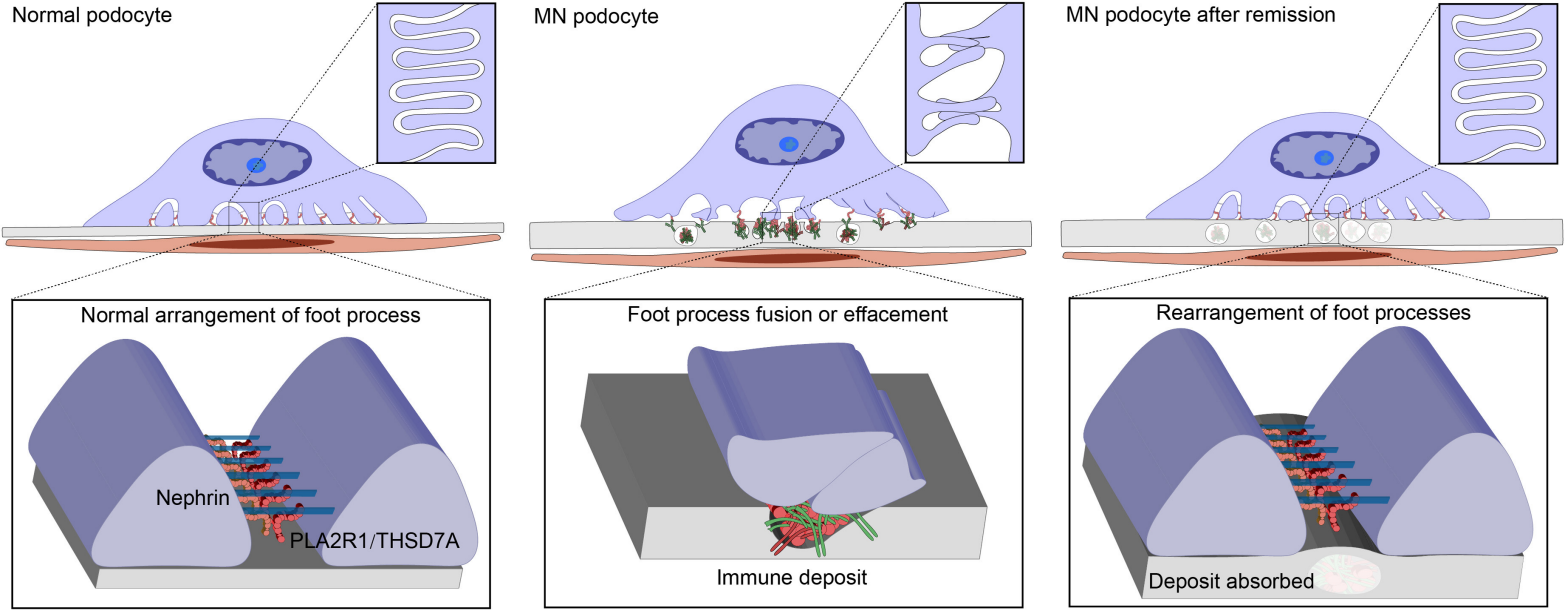
Changes of Podocyte morphology under various conditions. M-type receptor for secretory phospholipase A2 (PLA2R1) or thrombospondin type-1 domain-containing 7A (THSD7A) potentially maintain the slit diaphragm or the adherence of podocytes to glomerular basement membrane (GBM), involved in the normal arrangement of the foot processes. Anti-PLA2R1/THSD7A antibodies bind podocytes forming the immune deposits and resulted in thickness of GBM and morphological changes of podocytes, including cell body enlargement and foot process effacement. Under the damage degree that does not exceed the self-repairing ability threshold, the podocytes gradually goes toward self-healing with the gradual relief of pressure, recovering the original structure, and function. Foot processes rearrange and immune deposits are absorbed by GBM, and finally is the restoration of normal filtration capacity and proteinuria remission.

The podocyte slit-diaphragm between adjacent foot processes is the final filtration barrier of the glomerulus (112). Condensation of the actin cytoskeleton at the base of effaced podocyte foot processes is a prominent feature of both human and experimental MN, which is accompanied by various alterations in the intervening filtration slits, including widening, formation of occluding-type junctions, and displacement and disruption of slit-diaphragms (113). These changes are accompanied by a reduction in the amount and alteration in the distribution of nephrin and podocin, both of which are essential for slit-diaphragm integrity (114, 115). The slit-diaphragm is a multicomponent structure that includes heterophilic and homophilic interactions between nephrin, and neph 1, and possibly cadherins (112). Podocin is associated with the cytoplasmic tail of nephrin, and likely stabilizes nephrin and the slit-diaphragm in a lipid raft domain in the podocyte plasma membrane (116). As nephrin dissociates from actin and podocin, its ability to tether the cytoskeleton and plasma membrane gradually diminishes, which could be the cause for the dislocation of the slit diaphragm and proteinuria in rat PHN (115). The sublethal injury mediated by anti-Fx1A and complement *in vitro* was accompanied by the dissolution of F-actin microfilaments and loss of the focal adhesion complexes in rat podocytes. The cytoskeleton is dissociated from the matrix-attached integrins due to the loss of the focal adhesion complexes that anchors the cytoskeleton to the integrin. However, podocytes retained stromal integrins even in the case of injury (117). This finding corresponds to human's MN, and the distribution of β1-integrin in renal biopsies in patients with various glomerular diseases (including membranous and other forms of glomerulonephritis, minimal change disease) are similar to that in normal glomeruli (118). These findings can be related to the foot process fusion or effacement in MN. It is worth noting that, after the removal of anti-Fx1A and complement for 18 h, podocytes recover back to normal cellular morphology, indicating that the injury was reversible and podocytes have a certain level of self-repair ability (117). The glomerulus does have a self-healing ability depending upon the pressure applied; if the pressure remains within the limits of the self-repairing ability, the glomerulus gradually goes toward the self-healing process with the gradual relief of pressure, recovering its original function, and finally achieving the remission of proteinuria (119). In a pathological state, the specific marker proteins of podocytes gradually disappear, while macrophage-related markers, such as CD68, are gradually expressed (120). Podocytes mainly perform the important function of acting as a filtering barrier under normal circumstances; while undergoing phenotypic changes, podocytes can protect themselves under pressure. When the adverse factors are removed gradually, the podocytes transformed into normal phenotypes and resume a normal morphological structure, which could be the potential cause of proteinuria remission (Figure 4).

## Conclusions

In this review, we proposed a hypothetical model of IMN pathogenesis that extrarenal immune activity causes additional kidney damage, which could to some extent explain some clinical features of IMN. Environmental stimuli or other factors induce the exposure of auto-antigens to extrarenal tissues, and initiate the autoimmune response targeted podocytes. Most IMN patients have a common immune pathway characterized by IgG4 response, which may be the result of an extrarenal autoimmune response transition to anti-inflammatory. The anti-inflammatory properties of autoimmune responses may decorate the unique pathological pattern and renal limitation of IMN. The production of IgG4 outside the kidney promoted immune remission, and the reversibility of podocytes is a necessary condition for proteinuria remission, both of which jointly promoted spontaneous remission. Figures 3, 4 are simplified illustrations of this understanding. We believe that our proposed hypothesis is beneficial to the further understanding of MN and future study in this field.

## Author Contributions

WL, CG, XT, and BL contributed to the conception and design of the review study. WL, CG, and HD wrote the first draft of the manuscript. YG, ZD, YZ, ZL, and ZF wrote sections of the manuscript. XT and BL discussed and revised the content of the review article. All authors contributed to manuscript revision, read and approved the submitted version.

## Conflict of Interest

The authors declare that the research was conducted in the absence of any commercial or financial relationships that could be construed as a potential conflict of interest.
